# Biomonitoring of Urinary Cotinine Concentrations Associated with Plasma Levels of Nicotine Metabolites after Daily Cigarette Smoking in a Male Japanese Population

**DOI:** 10.3390/ijerph7072953

**Published:** 2010-07-20

**Authors:** Taku Nagano, Makiko Shimizu, Kazuma Kiyotani, Tetsuya Kamataki, Ryohji Takano, Norie Murayama, Fumiaki Shono, Hiroshi Yamazaki

**Affiliations:** 1 Laboratory of Drug Metabolism and Pharmacokinetics, Showa Pharmaceutical University, Machida, Tokyo 194-8543, Japan; E-Mails: p0910@g.shoyaku.ac.jp (T.N.); shimizu@ac.shoyaku.ac.jp (M.S.); takano.r@jp.fujitsu.com (R.T.); muraya_n@ac.shoyaku.ac.jp (N.M.); 2 Hokkaido University, Sapporo 060-0182, Japan; E-Mails: kkiyotani@src.riken.jp (K.K); snc78123@nifty.com (T.K.); 3 Japan Chemical Industry Associations (JCIA), Tokyo 104-0033, Japan; E-Mail: fshono@jcia-net.or.jp (F.S.); 4 High Technology Research Center, Showa Pharmaceutical University, Machida, Tokyo 194-8543, Japan

**Keywords:** cytochrome P450, CYP2A6, 3′-hydroxycotinine, genetic polymorphism, smoking index, biomarker

## Abstract

Human biomonitoring of plasma and urinary levels of nicotine, cotinine, and 3′-hydroxycotinine was conducted after daily cigarette smoking in a population of 92 male Japanese smokers with a mean age of 37 years who had smoked an average of 23 cigarettes per day for 16 years. Members of the population were genotyped for the nicotine-metabolizing enzyme cytochrome P450 2A6 (*CYP2A6*). The mean levels of nicotine, the levels of its metabolites cotinine and 3′-hydroxycotinine, and the sum of these three levels in subjects one hour after smoking the first cigarette on the sampling day were 20.1, 158, 27.7, and 198 ng/mL in plasma and 846, 1,020, 1,010, and 2,870 ng/mL in urine under daily smoking conditions. Plasma levels of 3′-hydroxycotinine and urinary levels of nicotine and 3′-hydroxycotinine were dependent on the CYP2A6 phenotype group, which was estimated from the *CYP2A6* genotypes of the subjects, including those with whole gene deletion. Plasma cotinine levels were significantly correlated with the number of cigarettes smoked on the day before sampling (*r* = 0.71), the average number of cigarettes smoked daily (*r* = 0.58), and the Brinkman index (daily cigarettes × years, *r* = 0.48) under the present conditions. The sum of nicotine, cotinine, and 3′-hydroxycotinine concentrations in plasma showed a similar relationship to that of the plasma cotinine levels. Urinary concentrations of cotinine and the sum of nicotine metabolite concentrations also showed significant correlations with the plasma levels and the previous day’s and average cigarette consumption. The numbers of cigarettes smoked per day by two subjects with self-reported light smoking habits were predicted by measuring the urinary cotinine concentrations and using linear regression equations derived from above-mentioned data. These results indicate that biomonitoring of the urinary cotinine concentration is a good, easy-to-use marker for plasma levels of cotinine and the sum of nicotine metabolites in smokers independent of genetic polymorphism of *CYP2A6*.

## 1. Introduction

Conventional cigarette smoking and environmental tobacco smoke have significant health effects [1–4]. Smoking is considered to cause cancer, stroke, and heart disease and also to have a close relationship with the occurrence of gastric ulcers, periodontal disease, sudden infant death syndrome, and metabolic syndrome [5–8]. Levels of cotinine, a metabolite of nicotine [9–11], in the blood track with exposure to tobacco smoke [1]. Promoting smoking cessation should therefore be a major priority in all countries [12] and for all health professionals in all clinical settings; however, it has been pointed out that in 2007 39.9% of Japanese men were current smokers [13]. It has been reported that even light smokers (1–10 cigarettes/day) have some increased risk of adult mortality [14]. Against this background, it has been reported that blood cotinine levels for nonsmokers in the United States population have decreased by about 70% in the past 15 years, indicating that public health interventions to reduce exposure have been successful (http://www.cdc.gov/exposurereport/), but such information in other groups or countries is limited [15,16]. Nicotine metabolite (cotinine and 3′-hydroxycotinine) concentrations in plasma and/or expired carbon monoxide have been examined as validated biomarkers of self-reported cigarette numbers associated with nicotine-metabolizing enzymes cytochrome P450 2A6 (*CYP2A6*) and *CYP2B6* genotypes [17,18].

We reported that white blood cell counts are sensitive biomarkers for smoking exposures and time-dependent recovery in healthy volunteers, and such data might be useful in education and in monitoring cigarette smoking and cessation [3]. The aim of the present study was to carry out a biomonitoring approach using nicotine and its primary and secondary metabolites, which are widely used as biomarkers for tobacco smoke in Japanese males. We report herein that the biomonitoring of cotinine in the urine is a good, easy-to-use marker for plasma levels of the sum of nicotine metabolites in Japanese smokers independent of genetic polymorphism of nicotine-metabolizing enzyme, *CYP2A6*. The number of cigarettes smoked per day in subjects with light smoking habits could also be predicted by measuring the urinary cotinine concentrations and using linear regression equations established in this study.

## 2. Experimental Section

### 2.1. Nicotine and Cotinine Determinations in Biological Samples from Male Smokers

This study was approved by the ethics committee of Hokkaido University and Showa Pharmaceutical University. Nicotine, cotinine (Wako Pure Chemicals, Osaka, Japan), and 3′-hydroxycotinine (Sigma-Aldrich, St. Louis, MO, USA) were used as standards. A total of 92 male Japanese smokers were recruited and were asked to provide information on the numbers of cigarettes smoked daily (Table 1). In separate experiments, two male self-reported light smokers (Subjects A and B, 23 and 25 years old, respectively) were recruited in an attempt to predict the level of smoking based on the first cohort study. Blood and urine samples from individual subjects were obtained 1 hour after smoking the first cigarette of the morning and were diluted 10 times with water. Conjugated nicotine and its metabolites in biological fluid samples were treated with β-glucuronidase (Wako).

Nicotine and its metabolite concentrations in these samples were measured by a liquid chromatography/mass spectrometry (LC/MS) system [18]. An LCQ Duo mass analyzer (Thermo Fisher Scientific, Yokohama, Japan) equipped with Xcalibur software was operated in electrospray positive ionization mode and was directly coupled to an Agilent 1100 system (Agilent Technology, Tokyo, Japan) with an octadecylsilane column (XBridge, 3.5 μm, 2.1 mm × 150 mm, Waters, Tokyo, Japan). To tune the mass spectrometer, the cone voltage was optimized to maximize the intensity of the precursor ions for nicotine (*m/z =* 163), cotinine (*m/z* = 177), and 3′-hydroxycotinine (*m/z* = 193). The collision energy was then adjusted to optimize the signal. Typical tuning conditions were as follow: capillary temperature, 225 °C; cone voltage, 25 V; ion spray voltage, 4.5 kV; and collision energy for nicotine, cotinine, and 3′-hydroxycotinine, 28, 30, and 30 kV, respectively, at sheath and aux gas (N_2_) flow rates of 35 and 5 arbitrary units, respectively. The gradient mobile phase consisted of 0.01% (v/v) ammonia and methanol; 0–0.5 min with 5% methanol (v/v) in 0.01% (v/v) ammonia; 0.5–3.0 min with 5%–16% methanol (v/v); 3–6 min with 16%–90% methanol (v/v); 6–12 min with 90%–5% methanol (v/v); 12–17 min with 5% methanol (v/v), at a flow rate of 0.20 mL/min.

### 2.2. CYP2A6 Genotyping

Genotyping of *CYP2A6* was carried out using genomic DNA isolated from blood samples [19,20]. Subjects were classified into four phenotype groups according to the *CYP2A6* genotypes. Group 1 was made up of subjects carrying CYP2A6*1/*1 (wild-type). Group 2 contained subjects heterozygous for the wild-type allele (CYP2A6*1/*4, CYP2A6*1/*7, CYP2A6*1/*9, or CYP2A6*1/*10). Group 3 consisted of subjects carrying CYP2A6*4/*7, CYP2A6*4/*9, CYP2A6*4/*10, CYP2A6*7/*7, CYP2A6*7/*9, CYP2A6*7/*10, CYP2A6*9/*9, CYP2A6*9/*10, or CYP2A6*10/*10. Group 4 contained subjects homozygous for the *CYP2A6* deletion allele (CYP2A6*4/*4), according to our previous reports [20,21].

## 3. Results and Discussion

**Figure 1:**
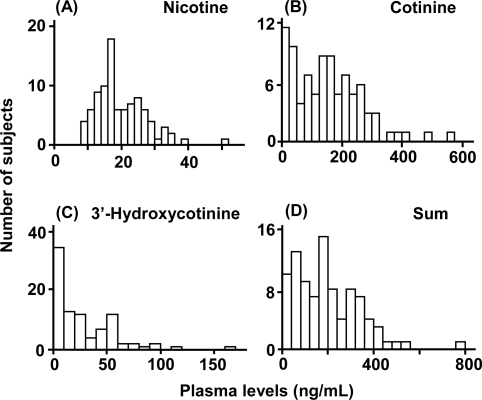
Plasma levels of nicotine and its metabolites in 92 male Japanese smokers 1 hour after smoking the first cigarette on the sampling day. Concentrations of nicotine (A), cotinine (B), and 3′-hydroxycotinine (C) were determined by LC/MS/MS. The sum (D) indicates the total concentration of nicotine, cotinine, and 3′-hydroxycotinine in each individual subject. Concentrations of 3′-hydroxycotinine were lower than the limit of detection in seven subjects.

**Figure 2:**
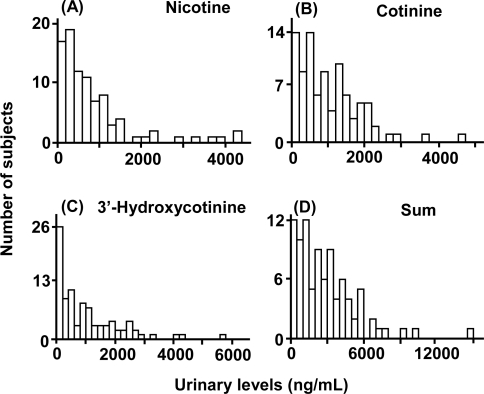
Urinary levels of nicotine and its metabolites in 91 male Japanese subjects 1 hour after smoking the first cigarette on the sampling day. Concentrations of nicotine (A), cotinine (B), and 3′-hydroxycotinine (C) were determined by LC/MS/MS. The sum (D) indicates the total concentration of nicotine, cotinine, and 3′-hydroxycotinine in each individual subject. A urine sample was not obtained from one subject.

The subject population consisted of 92 male Japanese smokers with a mean age of 37 years who had smoked an average of 23 cigarettes per day for 16 years (Table 1). Histograms of the concentrations of nicotine and its metabolites in plasma samples taken from these smokers one hour after smoking the first cigarette on the sampling day are shown in Figure 1. From the same data, the mean plasma levels of nicotine, its metabolites cotinine and 3′-hydroxycotinine, and the sum of the levels of these three compounds in the study population were 20.1, 158, 27.7, and 198 ng/mL, respectively (Table 2). The 95th percentile values of nicotine, cotinine, 3′-hydroxycotinine, and the sum were 33.5, 374, 89.1, and 446 ng/mL, respectively. Similar histograms of nicotine and its metabolite concentrations in urine samples are shown in Figure 2 at 1 hour after smoking the first cigarette on the sampling day; the mean and 95th percentile values of nicotine, cotinine, 3′-hydroxycotinine, and the sum were 846, 1,020, 1,010, and 2,870 ng/mL, and 3,540, 2,450, 3,060, and 7,470 ng/mL, respectively (Table 2).

**Figure 3:**
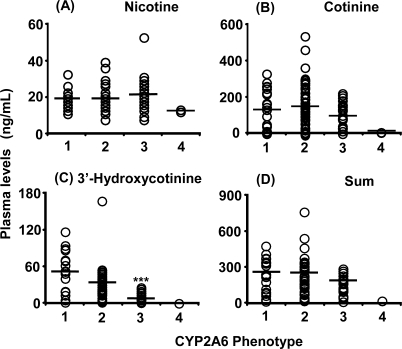
Plasma levels of nicotine and its metabolites in male Japanese smokers phenotyped for the *CYP2A6* gene. Individuals were grouped in either group 1, CYP2A6*1/*1 (n = 19); group 2, CYP2A6*1/*4, *7, *9, *10 (n = 42); group 3, CYP2A6*4, *7, *9, *10/*7, *9, *10 (n = 24); and group 4, CYP2A6*4/*4 (n = 2). Information on phenotype was not obtained from five subjects. Circles represent individual data and lines denote group means. ***, Significantly different compared with group 1 (*p* < 0.001).

**Figure 4:**
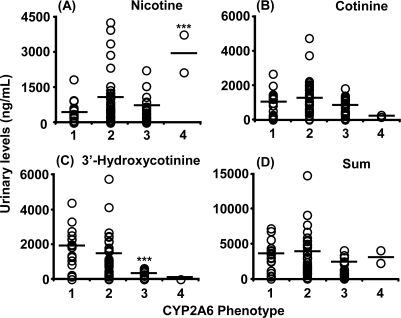
Urinary levels of nicotine and its metabolites in male Japanese smokers phenotyped for the *CYP2A6* gene. See legend of Figure 3 for further details.

**Figure 5:**
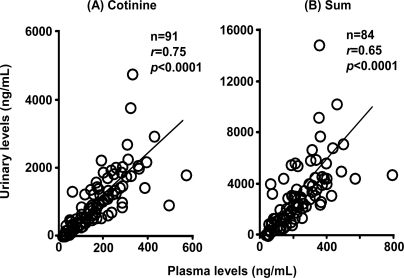
Correlation between individual urinary and plasma levels of cotinine (A) and the sum (B).

Plasma levels of 3′-hydroxycotinine (Figure 3C) and urinary levels of nicotine (Figure 4A) and 3′-hydroxycotinine (Figure 4C) were dependent on the *CYP2A6* phenotype group estimated from the subjects’ *CYP2A6* genotypes, including those with impaired function or whole gene deletion mutations. Concentrations of the secondary metabolite 3′-hydroxycotinine in plasma and urine were significantly lower in expected poor metabolizers; however, urinary unmetabolized nicotine levels in the same group were high. Cotinine levels in plasma (Figure 3B) and urine (Figure 4B) samples were not affected by the *CYP2A6* phenotype under daily cigarette smoking conditions. The sum of the metabolite concentrations in plasma (Figure 3D) and in urine (Figure 4D) were also independent of the *CYP2A6* phenotype. Urinary concentrations of cotinine and the sum of nicotine metabolites also showed significant correlations (*r* = 0.75 and 0.65) with their plasma levels (Figure 5), indicating that urinary cotinine concentration was a good marker for plasma levels of nicotine metabolites.

**Figure 6:**
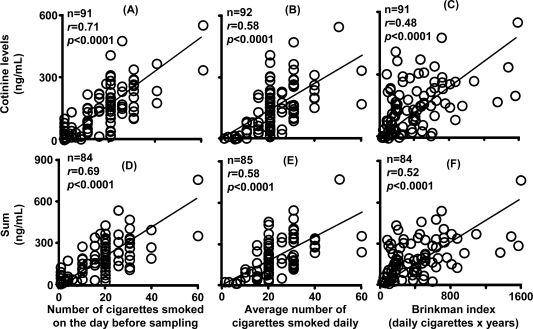
Relationship between plasma levels of cotinine (A,B,C) and sum (D,E,F) and three kinds of smoking status indicators. Information on the number of cigarettes smoked on the day before sampling and the Brinkman index was not obtained from one subject. The solid line represents the linear regression.

Plasma cotinine levels were significantly correlated with the number of cigarettes smoked on the day before sampling (Figure 6A, *r* = 0.71), the average number of cigarettes smoked per day (Figure 6B, *r* = 0.58), and the Brinkman index (Figure 6C, *r* = 0.48) under the present conditions (n = 91–92).

**Figure 7:**
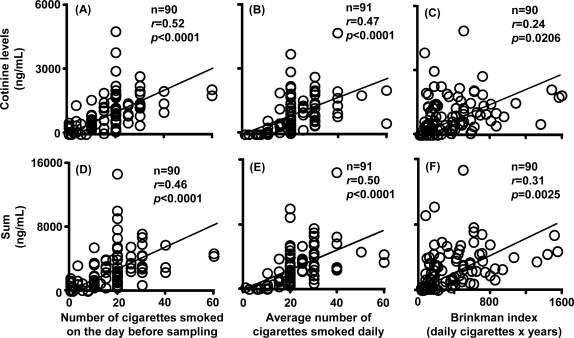
Relationship between urinary levels of cotinine (A,B,C) and sum (D,E,F) and three kinds of smoking status indicators. See legend of Figure 6 for further details.

The sum of nicotine, cotinine, and 3′-hydroxycotinine concentrations in plasma showed similar relationships (Figures 6D–F, *r* = 0.52–0.69). The relationship between urinary concentrations of cotinine showed low but significant correlation coefficients with the number of cigarettes smoked on the day before sampling (Figure 7A, *r* = 0.52), the average number of cigarettes smoked per day (Figure 7B, *r* = 0.47), and the Brinkman index (Figure 7C, *r* = 0.24) under the present conditions (n = 91–92). Similar correlation coefficients were seen for the sum of nicotine metabolites (Figures 7D–F).

Urinary cotinine excretion in self-reported light smokers was also measured (Table 3). From the slopes of the good linear regression lines shown in Figures 5A and 5B, plasma cotinine levels and the sum of nicotine metabolites were estimated by dividing the urinary cotinine concentration by 6.13 (*r* = 0.75) or the sum of metabolite concentrations in urine by 13.7. The number of cigarettes smoked on the previous day was then predicted by dividing the estimated plasma cotinine level by 8.24 (the slope in Figure 6A, *r* = 0.71) or the sum of metabolites in plasma by 10.4 (the slope in Figure 6D). When the urinary cotinine concentration was used, the actual number of cigarettes smoked on the preceding day as obtained by interview was able to be predicted using the positive relationships found in the present study.

It has been reported that single cigarette smoking might be a tool for *CYP2A6* phenotyping in those affected by loss-of-function gene mutations [22]. However, under daily smoking conditions, cumulative cotinine concentrations in the urine or plasma may not be affected by the *CYP2A6* genotypes, as shown in Figures 3B and 4B, which is consistent with our findings that nicotine could be metabolized by CYP2A6 and CYP2B6 in human liver microsomes [23]. Roles of exptrahepatic CYP2A13 [24,25] could not be ruled out in this nicotine metabolism. Significant, but relatively low correlation coefficients between the concentration of nicotine metabolites in biological fluids and the Brinkman index (number of cigarettes smoked × years) were also seen in the present study (Figures 6 and 7). Cotinine levels may be the best current biomarker for cigarette smoking [16] or secondhand smoke exposure [12], as reported previously.

## 4. Conclusions

Smoking is one of the most important behavioral risk factors for premature death worldwide [14]. The present results indicate that biomonitoring of the urinary cotinine concentration is a good, easy-to-use marker for plasma levels of cotinine and is convenient for predicting the number of cigarettes smoked on the preceding day under daily smoking conditions, independent of genetic polymorphism of *CYP2A6*.

## Figures and Tables

**Table 1:** Characteristics of 92 male Japanese smokers and their smoking behavior.

	**Mean (95% CI)**	**Median**	**95th percentile**	**Range**
Age	37 (35–39)	38	51	25–57
Number of cigarettes smoked on the day before sampling	17 (15–20)	20	40	0–60
Average number of cigarettes smoked per day	23 (21–25)	20	40	0.5–60
Brinkman index (daily cigarettes × years)	382 (312–452)	300	1160	7–1550
Years of smoking	16 (14–17)	16	31	2–38

**Table 2:** Plasma and urine levels of nicotine and its metabolites in 92 male Japanese subjects 1 hour after smoking the first cigarette of the day.

	**Mean (95% CI) (ng/mL)**	**Median (ng/mL)**	**95th percentile (ng/mL)**	**Range (ng/mL)**
Plasma levels				
Nicotine	20.1 (18.5–21.7)	17.8	33.5	8.10–51.8
Cotinine	158 (134–182)	146	374	3.52–556
3′-Hydroxycotinine	27.7 (21.6–33.7)	18.7	89.1	< 1–168
Sum a	198 (169–227)	183	446	15.0–763
Urine levels				
Nicotine	846 (648–1040)	561	3540	15.7–4400
Cotinine	1,020 (837–1120)	862	2450	15.8–4780
3′-Hydroxycotinine	1,010 (783–1240)	644	3060	3.84–5780
Sum a	2,870 (2350–3340)	2420	7470	46.8–14800

a Nicotine, cotinine, and 3′-hydroxycotinine concentrations were combined for individual subjects.

**Table 3:** Estimated number of cigarettes smoked on the day before sampling by biomonitoring of urinary cotinine concentrations in two subjects who were light smokers.

	**Subject**	
	**A**	**B**
Urinary concentration (ng/mL)		
Cotinine	136	396
Nicotine	79	29
3′-Hydroxycotinine	25	432
Sum	240	857
Number of cigarettes smoked on the day before sampling		
Predicted numbers based on cotinine concentration a	3	8
Predicted numbers based on the sum of concentrations b	2	6
Interviewed (self-reported) numbers	3	10

a From the slope of the linear regression line shown in Figure 5A, the plasma cotinine level was estimated by dividing the urinary cotinine concentration by 6.13. The number of cigarettes smoked on the day before sampling was predicted by dividing the estimated plasma cotinine level by 8.24 (the slope in Figure 6A).

b From the slope of the linear regression line shown in Figure 5B, the nicotine equivalent (sum) in plasma was calculated by dividing the sum in urine by 13.7. The number of cigarettes smoked on the day before sampling was predicted by dividing the estimated sum in plasma by 10.4 (the slope in Figure 6D).
